# INVESTIGATING THE RELATIONSHIP BETWEEN SOCIAL SUPPORT AND COGNITION AS MEDIATED BY HEALTH AND POSITIVE AFFECT

**DOI:** 10.1093/geroni/igz038.2407

**Published:** 2019-11-08

**Authors:** Francesca Falzarano, Karen L Siedlecki, Timothy Salthouse

**Affiliations:** 1 Fordham University, Bronx, New York, United States; 2 University of Virginia, Charlottesville, Virginia, United States

## Abstract

Decreased social networks are common in old age after major life events such as retirement, loss of loved ones, and declining health (Shankar et al., 2013). Diminished social ties are associated with increased feelings of loneliness and perceived isolation, which can have negative effects on cognition and physical health. The current study examines the relationship between social support (assessed via the Social Network Questionnaire) and overall cognitive performance (assessed as a latent construct comprising indicators that represent mean verbal episodic memory, processing speed, reasoning, and spatial visualization), and investigates positive affect and self-rated health as mediators of this relationship. The current study included 5,125 participants between the ages of 18-99 years from the Virginia Cognitive Aging Project (VCAP). Cross-sectional analyses were conducted using structural equation modeling. After controlling for age and education, results showed that a social support construct (comprising indicators representing each social network subscale) significantly and positively predicted cognitive performance (.59, p< .001). This relationship was reduced to .22 (p <.001) when positive affect was included as a mediator, and to .14 (p< .001) when self-rated health was included as a mediator. When the variables were included in a joint mediation model the relationship between social support and cognition was .20 (p < .001). Thus, health and positive affect are partial mediators of the relationship between social support and cognition and may help explain the relationship between social support and cognition. Furthermore, these findings provide additional evidence that social networks may play an important role in successful aging.

